# Cell Fusion May Be Involved in the Homothallic Mating of *Pneumocystis* Species

**DOI:** 10.1128/mbio.00859-22

**Published:** 2022-06-21

**Authors:** Philippe M. Hauser, Joao M. G. C. F. Almeida, Sophie Richard, Caroline S. Meier

**Affiliations:** a Institute of Microbiology, Lausanne University Hospital and University of Lausanne, Lausanne, Switzerland; b UCIBIO—REQUIMTE, Faculdade de Ciências e Tecnologia, Universidade Nova de Lisboa, Caparica, Portugal; Duke University

**Keywords:** genetic diversity, mycosis, nuclear fusion, obligate biotrophic parasite, sexual development, trophic form

## Abstract

*Pneumocystis* species are obligate fungal biotrophs that colonize the lungs of mammals. They cause deadly pneumonia in immunocompromised hosts. The sexual phase seems obligate during their life cycle and essential for survival because it is believed to ensure proliferation and transmission between hosts. Here, we consider if the sexual phase is initiated by the fusion of two cells or by nucleus duplication in order to generate diploid cells that can undergo meiosis. The juxtaposition of the nucleus-associated organelles of pairs of cells with fused cytoplasmic membranes demonstrated that cell fusion can occur. Nevertheless, the frequency of cell fusion remains to be determined, and it cannot be excluded that both cell fusion and nucleus duplication are used to ensure the occurrence of the essential sexual phase. *In vitro* culturing of these fungi is a major milestone that could clarify the issue.

## OPINION/HYPOTHESIS

The fungal genus *Pneumocystis* encompasses a myriad of species that colonize the lungs of mammals (1). Each *Pneumocystis* species is specific to a single mammalian species, although exceptions exist in small mammals (2). Genome sequencing revealed the obligate parasitism of these fungi because of the absence of many genes required for essential metabolic pathways. These fungi might be the only known obligate biotrophic parasites infecting animals, a type of parasite that is commonly observed in plants (3). Should the host immune system fail, they can turn into opportunistic pathogens that cause life-threatening pneumonia. The one caused by Pneumocystis jirovecii in humans is among the most frequent fungal invasive infections (4) and, thus, a public health issue.

Despite decades of efforts, no reproducible method for long-term *in vitro* culture for *Pneumocystis* species has been described. The reluctance to grow *in vitro* is a hallmark of obligate biotrophs (3). Consequently, the life cycle of these fungi cannot be directly observed and studied in the laboratory, and only hypotheses concerning its processes can be drawn. The proposed life cycles consisted of two phases: the asexual reproduction of trophic cells by binary fission or by “endogeny” (a poorly characterized generation of daughter cells within the cytoplasm of a single cell) and the sexual reproduction initiated by the mating of two trophic cells through their fusion (5–7). The sexual cycle culminates by the generation of an ascus that is thought to be involved in the airborne propagation to other hosts, as well as in the proliferation of the fungus within the host lungs (7, 8). Thus, sexuality is essential for the survival of the fungus, and, consistently, it was found to be obligatory during infection because the sex-related genes are concomitantly expressed and asci are always present. Obligate sexuality is also observed in plant obligate biotrophs that complete their entire cell cycle within their hosts (3, 9). Primary homothallism was found to be the mode of sexual reproduction adopted by *Pneumocystis* species (10). This mode involves a single self-compatible type that can undergo meiosis on its own, and it is common among fungi (11). Such sexuality is governed by a mating-type locus that includes genes responsible for the differentiation into both plus and minus mating types. Thus, each cell is of both mating types at the same time. Primary homothallism contrasts with heterothallism involving independent and compatible mating partners, each with either plus or minus identity. For pathogenic fungi, it is postulated that this mode is favored in restricted niches because it alleviates the need to find a compatible mating partner while keeping the advantages of sex, i.e., the increase of genetic diversity and virulence and the elimination of deleterious mutations (12). In contrast, the occurrence of the asexual cycle is debated (8). It could be involved in latency for survival and may predominate at the beginning of infection or in particular host conditions such as loss of thymus, immune reconstitution, or prophylaxis breakthrough. The data suggest that the asexual cycle may not contribute to the proliferation of the fungus during growth but might be facultative.

**FIG 1:**
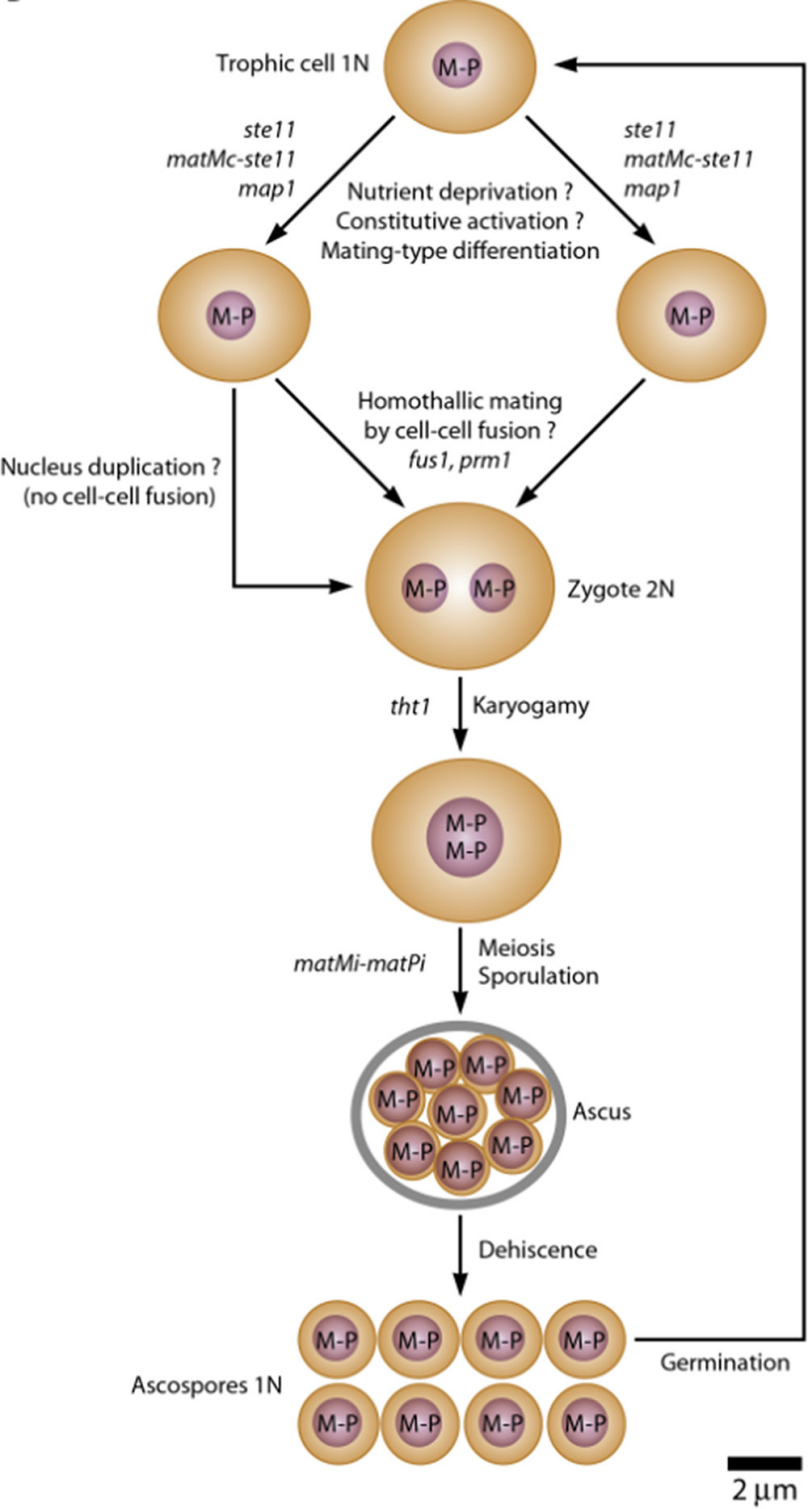
Hypothetical primary homothallism of *Pneumocystis* species. The question marks indicate events that are not demonstrated. The occurrence or not of homothallic mating by cell-cell fusion remains to be determined. The involvement of the genes mentioned is presumed but not established. M-P, fused plus and minus MAT loci on the same chromosome. The present article concludes that this model is valid, except the question mark for cell fusion. This figure is reprinted from Fig. 2B in reference 9 with permission.

The question arose whether fusion of trophic cells during sexual cycle generates diploid cells that are able to enter into meiosis, as assumed in the models, or if a nucleus duplication replaces cell fusion (9) (Fig. 1). This question was prompted by the two following reasons.

(i) Most studies interpreted pairs of trophic cells with fused cytoplasmic membranes as cell division rather than cell fusion, without giving explicit arguments.
(ii) *Pneumocystis*’s relatives of the subphylum Taphrinomycotina rely either on cell fusion or on nucleus duplication. Cell fusion was reported in the genera *Schizosaccharomyces* (13) and *Protomyces* (14), whereas nucleus duplication takes place in the genera *Neolecta* (15) and *Taphrina* (16). Nucleus duplication in *Taphrina* is relevant because this genus would rely on primary homothallism like *Pneumocystis* (10). Indeed, this mechanism is possible in primary homothallism because a compatible partner is not necessary for mating, i.e., each strain can enter into the sexual cycle on its own.

The question whether cell fusion occurs during sexuality is crucial in order to understand how genetic diversity is generated during the life cycle and evolution of *Pneumocystis* species. Cell fusion allows a substantial increase of this parameter through possible mating of different strains with distinct genetic backgrounds (outcrossing). In the present article, we review the available data and conclude that cell fusion can occur during the sexual cycle of *Pneumocystis* species, but its frequency remains to be determined.

## MORPHOLOGICAL EVIDENCE OF FUSION OF TROPHIC CELLS

**FIG 2:**
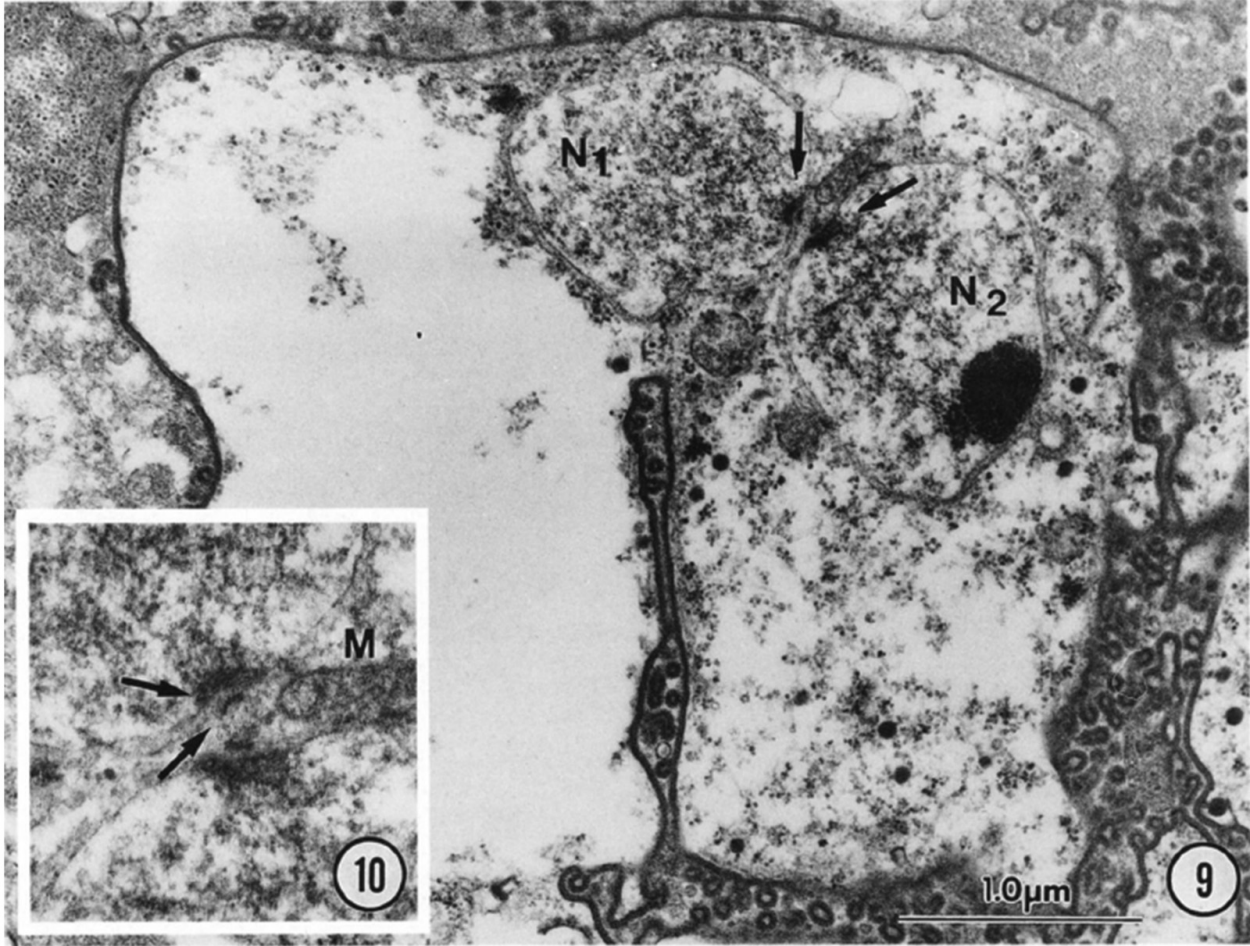
Binucleated P. carinii trophic form suggestive of conjugation. Panel 9, nuclei (N1 and N2) with intact nuclear envelopes are positioned with NAO in direct apposition (arrows). Panel 10, higher magnification of NAO (2× panel 9). Arrows indicate two-part construction of NAO with closely associated mitochondrion (M). The two panels and their legend are Fig. 9 and 10 of Itatani (17). Republished with permission of the Journal of Parasitology, Allen Press Publishing Services.

**FIG 3:**
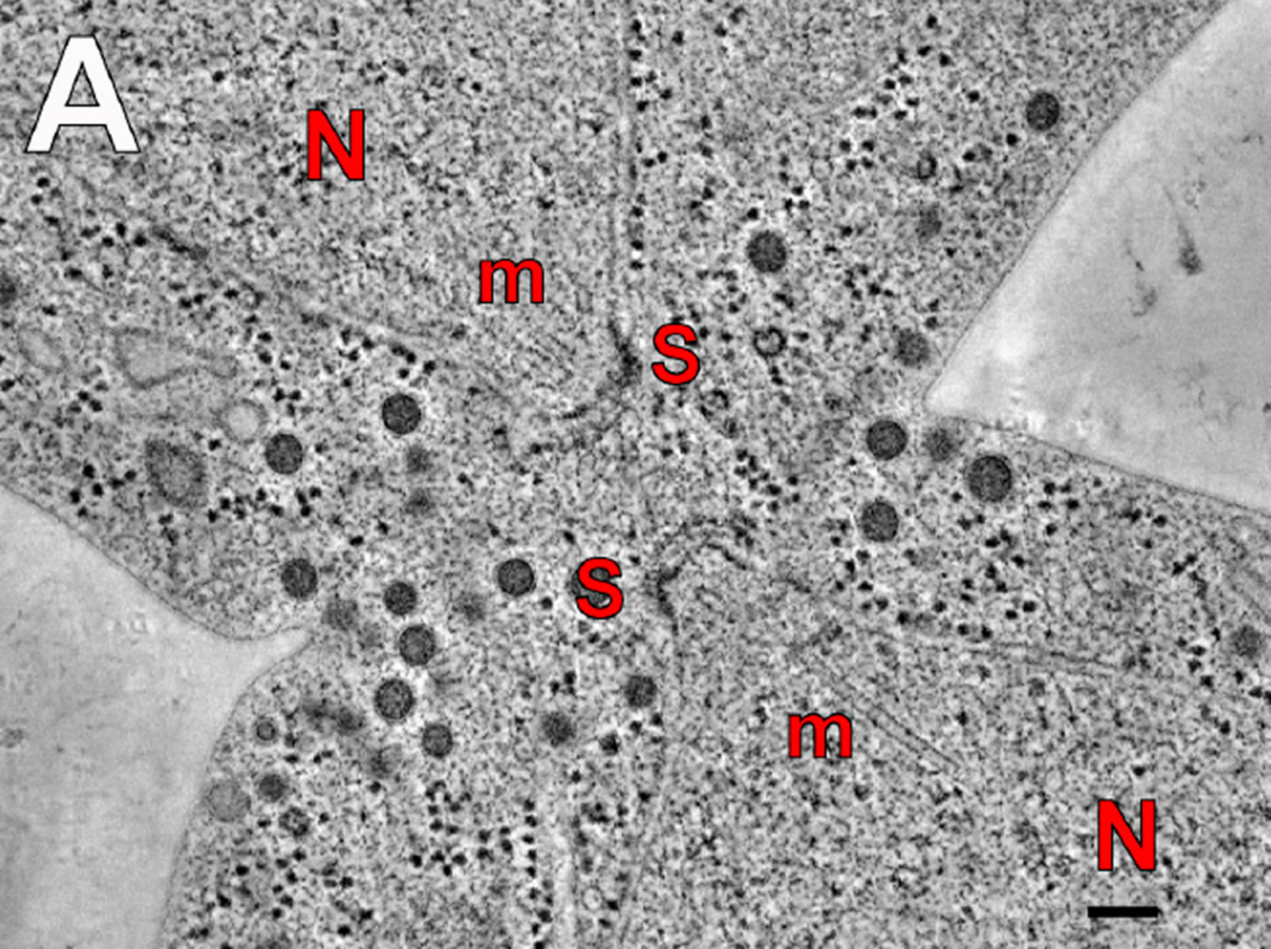
A slice through a tomogram of wild-type mating in which cell fusion but not nuclear fusion has occurred. N, nucleus; m, microtubule; S, SPB (=NAO); bar, 100 nm. This figure is reprinted from Fig. 3A in reference 19 with permission from Rockefeller University Press.

In the absence of *in vitro* culture, the study of the life cycle of *Pneumocystis* species relies mainly on electron microscope images of resected lungs infected with *Pneumocystis*, commonly from rodents. The studies were not quantitative, and only example images were published, so the frequency of each recognized stage or process is not known. Among circa 10 studies, only one reported morphological structures that demonstrate the occurrence of cell fusion (17). Two pairs of trophic cells of Pneumocystis carinii infecting rats show fusion of their cellular membranes and their nuclei close to each other. The two nuclei are located within the isthmus presumably produced by the cell fusion and have their nucleus-associated organelle (NAO) in close apposition (Fig. 2). This apposition and the location within the isthmus constitute a hallmark of cell fusion during sexual mating that was characterized in the close relatives Schizosaccharomyces pombe (18) and Saccharomyces cerevisiae (19) (Fig. 3). NAOs are believed to draw nuclei toward each other through attachment to microtubules, before they fuse.

Itatani suggested a further hallmark of cell fusion on her two images: the presence of a single indentation of the fused cellular membrane (Fig. 2). This would result from cell fusion starting from the single point of contact between the two fusing trophic cells. It would contrast from constriction of the membrane during cell division that creates two symmetrical indentations. The curved shape of the zygote resembles that observed in zygotes of Schizosaccharomyces octosporus (20) and S. pombe (13), suggesting a related phenomenon. However, the single indentation phenomenon has been reported neither in these two species nor in S. cerevisiae (19). The absence in the latter yeast might be related to the fact that mating involves shmooing before cell fusion, i.e., cytoplasmic extension toward the sexual partner. The latter phenomenon has not been observed in *Pneumocystis*. Three other images presented similar pairs of *Pneumocystis* trophic cells with fused cytoplasmic membranes and a single indentation of the membrane but without NAOs (5, 21). Although the hallmark consisting in the single indentation appears possible, it is an argument in favor of cell fusion that remains to be confirmed.

In conclusion, Itatani’s images demonstrate that cell fusion can be involved in the sexual cycle of P. carinii infecting rats. Surprisingly, only few articles mentioned this observation (7, 22, 23). The rarity of such images might reflect that the process is fast, so only a small proportion of the cells are fusing at a given time. However, this is not suggested by the long generation time of P. carinii *in vivo*, i.e., 4.5 days (24). Alternatively, it might be a rare event, highlighting that the frequency of cell fusion remains to be determined.

## PAIRS OF TROPHIC FORMS WITH FUSED CYTOPLASMIC MEMBRANES

**FIG 4:**
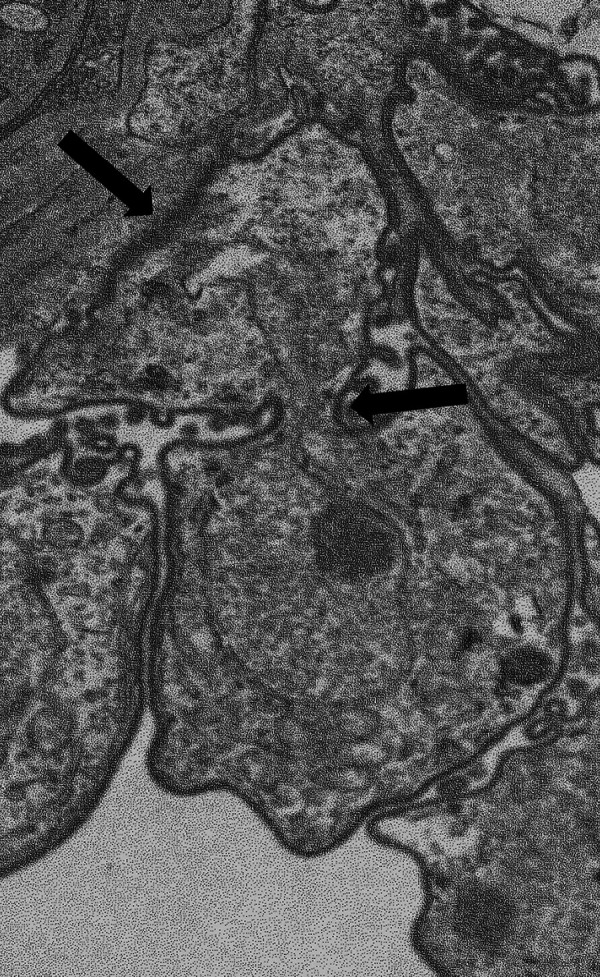
A trophic form tightly interdigitated with the host’s alveolar epithelial pneumocytes type I (top arrow). This trophic form also shows fusion with a second trophic form at the level of the cell membrane as well as possibly at the level of the nuclear membrane (middle arrow). This figure is reprinted from Fig. 2A of reference 8 under the terms of the Creative Commons Public Domain declaration (https://creativecommons.org/publicdomain/zero/1.0).

Seventeen images of two trophic cells showing fusion of their cytoplasmic membranes were published (5, 6, 8, 17, 21, 25, 26). Three of them also showed fusion of their nuclear membranes (5, 8, 26); an example is shown in Fig. 4. These pairs may correspond to cell fusion or division, whereas budding or other asymmetric division processes are unlikely because the cells involved were always of similar size. Twelve of the 17 pairs were interpreted as cell division, without providing explicit arguments. The remaining five were not interpreted because it was considered that they could correspond to either a cell division or a cell fusion (5, 6, 8, 17). We fully agree with the latter caveat. It results from the static nature of the images as well as from the two following reasons.

(i) No evidence of sexual dimorphism in Pneumocystis species was reported so far; hence, isogamous mating of trophic cells would occur. Indeed, no type of cells other than trophic forms and asci that could participate in anisogamy was reported. Thus, the pairs of trophic cells with fused cytoplasmic membranes could correspond to cell fusions. The absence of juxtaposed NAOs in these pairs would be consistent with a more advanced stage of cell fusion than when NAOs are present, i.e. before nuclei fusion.
(ii) *Pneumocystis* species harbor most probably a closed or partially closed (semi-open) mitosis. These are modes of cell division where the nuclear membrane remains intact or is perforated during mitosis and meiosis. These modes are observed in the vast majority of fungal phyla (27), including Ascomycota, to which *Pneumocystis* belongs. Some members of the Basidiomycota are the only exceptions. They harbor an open mitosis like plants and animals, i.e., with a complete breakdown of the nuclear membrane when chromosomes segregate during metaphase and anaphase. The closed or partially closed mitosis implies that the reported pairs of trophic cells might correspond to a cell division despite the presence of fusion of their nuclear membranes.

According to Itatani’s hypothesis concerning the single indentation of the membrane during sexual mating, 13 of the 17 pairs of trophic cells with fused membranes might correspond to a cell division rather than a cell fusion because they present two symmetrical indentations. However, this remains a weak argument because the single indentation might not be observed or even occur at each cell fusion. No microtubule spindles involved in the segregation of the chromosomes, which is a hallmark of nuclear and cell division, were present in the published pairs of trophic cells with fused membranes. This absence does not exclude cell division because constriction of the membranes to produce two separate cells occurs after the formation of the spindle and chromosome segregation. Only three images of *Pneumocystis* cells showed such spindles within a single cell (5, 6, 28). Two of these nuclear divisions were interpreted as one of the two cell divisions that occur in young asci during meiosis I and II (5, 28). The last one was considered a cell division of trophic cells by binary fission or endogeny (6).

In conclusion, the observed pairs of trophic forms with fused cytoplasmic membranes could correspond to cell divisions, cell fusions, or a mixture of both processes. It is possible that they correspond in majority to cell fusions because the sexual cycle could be the main process contributing to the fungus proliferation.

## IS GENETICS COMPATIBLE WITH CELL FUSION?

The pathways involved in sexuality are highly diverse among fungi (11). They have been constantly rewired during fungal evolution. Consequently, the presence or absence of specific orthologous genes in a species does not allow concluding if a given process takes place. The sexuality of *Pneumocystis* might be initiated by sensing environmental parameters, as in other fungi, followed by activation of the transcription factor Ste11. The latter would in turn activate the genes of the mating-type locus and lead to cell differentiation for mating through the coexpression of both plus and minus genes in each cell (9). The genes encoding two receptors of the sexual pheromones are present in *Pneumocystis* genomes (10, 29), and fluorescence microscopy revealed that both receptors are presented at the surface of each trophic cell (30). This plays in favor of the occurrence of cell fusion because the interaction between pheromones and receptors generally precedes cell fusion in fungi. However, it does not constitute a proof because, for example, one such receptor gene is present in the Taphrina deformans genome despite the absence of cell fusion in its sexuality. Indeed, the T. deformans sexual cycle involves the fusion of the two nuclei of the diploid proascus, followed by meiosis and the development of the ascus (16). Moreover, in other primary homothallic fungi, these receptors are involved in postfertilization events rather than cell fusion (see reference 30 for references). *Pneumocystis* species harbor genes orthologous to those that are essential for cell fusion in the close relative Schizosaccharomyces pombe (*fus1*, *prm1*, *cfr1*) (13, 31). However, these genes are also present in *T. deformans* (10) and, thus, are possibly involved in processes other than cell fusion. The *tht1/kar5* gene, which is considered a signature of karyogamy (32), is present in *Pneumocystis* genomes (29). However, nucleus duplication could be followed by karyogamy of sister mitotic nuclei rather than by simple DNA duplication within a single nucleus. Thus, karyogamy might also occur in the absence of cell fusion, as it occurs in *T. deformans*.

In conclusion, the genes present in *Pneumocystis* genomes are compatible with the occurrence of cell fusion during sexuality but do not prove or suggest its occurrence. In the absence of culture *in vitro*, functional complementation in budding or fission yeast provides a means to test hypotheses.

## MITOCHONDRIAL HETEROPLASMY IS COMPATIBLE WITH CELL FUSION

Mitochondrial heteroplasmy in P. jirovecii infecting humans has been suggested on the basis of the diversity of the genomic markers of this organelle greater than that of the nuclear ones (33). This was assessed by the use of ultradeep sequencing that supported the presence of a single allele of nuclear genes in some isolates while containing several alleles of the mitochondrial genes. Mitochondrial heteroplasmy could result from (i) recombination by a homing endonuclease, as observed in many fungi (34), (ii) a frequency of mutations higher in the organelle than in the nucleus (33), or (iii) isogamic sexual mating by fusion of trophic cells because this would lead to biparental inheritance of the mitochondria. However, the latter hypothesis is not supported by the concomitant purity of the nuclear markers because cell fusion would lead also to their heterogeneity.

In conclusion, mitochondrial heteroplasmy might be frequent in *P. jirovecii* because of a homing endonuclease and/or a high frequency of mutations. The issue remains to be investigated in other *Pneumocystis* species.

## CONCLUSIONS

The observation of NAOs juxtaposed in two pairs of trophic forms with fused cytoplasmic membranes demonstrated that cell fusion can occur during the sexual mating of P. carinii. Nevertheless, we cannot exclude that cell fusion occurs only in P. carinii because no data are available for the other *Pneumocystis* species. Moreover, the frequency of cell fusion remains to be determined. The outcrossing resulting from cell fusion allows genetic variation and diversity that might be essential for biological adaptation of *Pneumocystis*, as in many other organisms. Although no other cases have been reported so far, it cannot be excluded that two mechanisms of diploidization are maintained in *Pneumocystis*, cell fusion and nucleus duplication. This could ensure the occurrence of sexuality in all circumstances as it is essential for *Pneumocystis*. In our opinion, maintaining two mechanisms of diploidization in parallel appears unlikely because it would not be economical because all trophic cells harbor the receptors of the two mating types (30). Indeed, all trophic cells should be able to fuse with any other trophic cell present in the infection and, thus, may not need a backup system for diploidization. However, other constraints may play a role, and further investigation is warranted. The model of the *Pneumocystis* sexual cycle shown in Fig. 1 remains valid, except that the occurrence of the cell fusion is likely. To clarify the issue, *in vitro* culturing would be ideal, but alternatives may help, such as short-term cell free culture, functional complementation assays, or culture within organoids (35). In addition, genetic transformation of *Pneumocystis* might be developed, as performed for the other obligate parasite *Chlamydia* (36). Differential fluorescent labeling of populations for testing the hypothesis of cell fusion in animal models could also be attempted.
